# Fault Diagnosis Method for High-Pressure Common Rail Injector Based on IFOA-VMD and Hierarchical Dispersion Entropy

**DOI:** 10.3390/e21100923

**Published:** 2019-09-23

**Authors:** Enzhe Song, Yun Ke, Chong Yao, Quan Dong, Liping Yang

**Affiliations:** Institute of Power and Energy Engineering, Harbin Engineering University, Harbin 150001, Heilongjiang, China; sez2005@hrbeu.edu.cn (E.S.); yaochong@hrbeu.edu.cn (C.Y.); dong_quan@hrbeu.edu.cn (Q.D.); yangliping302@hrbeu.edu.cn (L.Y.)

**Keywords:** variational mode decomposition, improved fruit fly optimization algorithm, hierarchical dispersion entropy, high-pressure common rail injector, fault diagnosis

## Abstract

The normal operation of high-pressure common rail injector is one of the important prerequisites for the healthy and reliable operation of diesel engines. Therefore, this paper studies the high-precision fault diagnosis method for injectors. Firstly, this paper chooses VMD to adaptively decompose the common rail fuel pressure wave. The biggest difficulty in VMD decomposition is the need to manually set the internal combination parameters K and α. In order to overcome this shortcoming, this paper proposes an improved fruit fly search. The variational mode decomposition method of the algorithm, with the energy growth factor e as the objective function, can adaptively decompose the multi-component signal into superimposed sub-signals. In addition, based on the analytic hierarchy process and dispersion entropy, hierarchical dispersion entropy is proposed to obtain a comprehensive and accurate complexity estimation of time series. Then, a fault diagnosis scheme for high-pressure common rail injector based on IFOA-VMD and HDE is proposed. Finally, using the engineering test data, the method is compared with other methods. The proposed method appears, based on the numerical examples, to be better from both a computational and classification accuracy point of view.

## 1. Introduction

As an important power machinery in production and life, high-pressure common rail diesel engine plays an indispensable role in various industries, and the normal operation of diesel engine is a solid guarantee for our rich life [1,2]. The diesel injector is a key component of the diesel engine, and its working state will directly affect the operating power of the diesel engine [3]. Because the injector works in the high-temperature and high-pressure environment inside the cylinder, faults occur frequently such as nozzle clogging, solenoid valve failure, needle valve stuck, etc. [4]. These failures of the injector may result in abnormal fuel injection and uneven operation of each cylinder and may even result in further reduction in combustion efficiency and more exhaust emissions. Therefore, it is very necessary to diagnose the injector of high-pressure common rail diesel engine. Because the vibration signal combines a variety of information, including gas depletion, combustion shock, structural vibration, noise, etc., resulting in complex data processing and low diagnostic accuracy of fault feature extraction, the fuel pressure in common rail pipe and high-pressure oil pipe can acquire directly injection process information in cylinder [5].

The injector operating status information can be reflected by the common rail fuel pressure wave, but the fuel pressure wave is a non-linear and non-stationary signal. In order to extract time domain and frequency domain information at the same time, it is necessary to apply joint time–frequency analysis method for fault detection [6,7,8]. Wigner–Ville distribution (WVD), wavelet transform (WT), empirical mode decomposition (EMD), and local average decomposition (LMD) are commonly used representative methods. However, each representative method has some inherent defects in the extraction of non-stationary signal information. For example, WVD has unavoidable cross-interference terms, which has become an obstacle to its widespread application in signal processing [9]. The wavelet transform can divide the frequency band into multiple layers, but it cannot further decompose the high frequency part, and the selection of the wavelet base has a significant influence on the decomposition effect, and the adaptive ability is insufficient [10]. The EMD method has the advantages of intuitiveness and a-posteriori adaptability, but the shortcomings of mode aliasing, end effect, envelope overshoot and undershoot cannot be solved well [11]. Compared with EMD, the Iterative Filtering method (IF) [12,13,14] and its fast implementation (Fast Iterative Filtering, FIF) based on the Fast Fourier Transform [15] has a solid mathematical background and all the advantages of the original EMD method and without the well-known shortcomings of mode aliasing, end effects, envelope overshoot and undershoot. The LMD method is an improvement of the EMD method, but when the original signal is disturbed by noise and the harmonic components with similar frequency components are decomposed, there are certain modal aliasing, end effect, and false component, which reduces the resolution and detection effect of the algorithm [16].

Dragomiretskiy and Zosso proposed a variational mode decomposition (VMD) processing algorithm in 2014 [17]. Unlike the traditional recursive algorithms of EMD, EEMD, and LMD (local mean decomposition), the VMD algorithm is free of the recursive screening and stripping mode of traditional signal decomposition. It can alleviate modal aliasing and boundary effects with a solid mathematical foundation, as well as the advantages of high-computational efficiency and robustness. However, VMD decomposition number K and α the penalty parameter α need to be preset, and inappropriate parameters may cause information loss or excessive decomposition problems, which may affect subsequent feature extraction [18,19,20]. In 2011, Wen-Tsao Pan was inspired by the foraging behavior of Drosophila, and thus proposed the Fruit Fly Optimization Algorithm (FOA) [21]. Compared with existing heuristic algorithms (such as genetic algorithm (GA), particle swarm optimization (PSO), and ant colony algorithm), FOA has few parameters to adjust, and it is easy to understand and implement by virtue of the simple computational process. Moreover, it has many advantages such as less control variables, strong control ability, and good global optimization. The optimization solves these problems and has been used by many scholars to solve complex parameter optimization problems [22,23,24]. However, when a fruit fly in the fruit fly population finds the optimal solution for this iteration, all the fruit flies gather at the location of the fruit fly, but if the fruit found by the fruit fly is not the global optimal solution, then the algorithm falls into local optimum. Based on this, this paper proposes a variational mode decomposition based on the improved fruit fly algorithm (IFOA), which can not only adaptively optimize the internal parameters K and α of VMD but also improve the convergence speed and convergence precision of the algorithm.

How to extract fault characteristic information from nonlinear time series is the key to fault diagnosis of high-pressure common rail injector. In recent years, with the development of nonlinear scientific theories, various information entropy methods have emerged. Pincus [25] proposed the concept of approximate entropy in 1991. Then, for the self-matching defect of approximate entropy, Richman et al. [26] proposed the concept of sample entropy. Sample entropy is a commonly used feature extraction method, which has the advantages of strong anti-noise ability and short time series, but the method fault feature states can only be described from a single scale. Costa et al. [27,28] proposed a multi-scale entropy (MSE) based on sample entropy to measure the complexity of time series at different scales. Aiming at the sample entropy similarity measure in MSE, the mutation occurs easily. Zheng et al. [29], combined with the concept of fuzzy entropy, proposed multiscale fuzzy entropy (MFE) and applied it to the fault diagnosis of rolling bearings. In order to extract the fault information of high-frequency components in the signal, Jiang Ying [30] introduced the concept of hierarchical fuzzy entropy. Compared with multi-scale fuzzy entropy, hierarchical fuzzy entropy considers both the low-frequency component and the high-frequency component of the signal, thus providing more comprehensive and accurate time mode information. In order to alleviate the shortcomings of sample entropy, fuzzy entropy, and permutation entropy, Azami [31] proposed dispersion entropy (DE) and proposed to measure the complexity of time series from different scale factors. Multiscale Dispersion Entropy (MDE) [32] does not need to rank the amplitude values of one embedded vector, nor does it need to calculate the distance between any two composite delay vectors in different embedding dimensions. Compared with sample entropy and fuzzy entropy, dispersion entropy has the advantages of simple and fast calculation [33,34]. At the same time, dispersion entropy overcomes the main defects of permutation entropy and effectively solves the influence of medium amplitude of embedding vector [35]. The study of analog signals and biosignals shows that dispersion entropy is relatively insensitive to noise and excellent in anti-interference compared to sample entropy and fuzzy entropy because small changes in amplitude values do not change their class labels; dispersion entropy is more sensitive to changes in the synchronization frequency, amplitude value, and signal bandwidth. Therefore, based on the superiority of analytic hierarchy process and dispersion entropy, hierarchical dispersion entropy (HDE) based on hierarchical entropy and dispersion entropy is proposed. The method describes the complexity and uncertainty of the sequence from different levels and reduces the deviation of the single scale, which realizes the feature richness of the signal sequence from many aspects. Compared with multi-scale sample entropy, hierarchical sample entropy [36], multi-scale fuzzy entropy, hierarchical fuzzy entropy, and multi-scale dispersion entropy, it can not only consider the high-frequency and low-frequency components of the original sequence but also improve the anti-interference and signal bandwidth variation sensitivity. Finally, combining the IFOA-VMD algorithm with hierarchical dispersion entropy, a new fault diagnosis method for high-pressure common rail injector is proposed and applied to the analysis of engineering test data.

The rest of this article is organized as follows. In Section 2, the principle of the VMD algorithm is briefly introduced and the flow of the IFOA-VMD algorithm is described. Then, in Section 3, the hierarchical dispersion entropy algorithm flow is described, and the effectiveness of the proposed method is verified by numerical simulation signals. In Section 4, the fuel pressure wave signal of the high-pressure common rail injector is analyzed by the fault diagnosis method proposed in this paper, and the effectiveness and superiority of the method are verified by SVM. Finally, the conclusion is given in Section 5.

## 2. Improved Adaptive VMD Algorithm

### 2.1. VMD Decomposition Principle

VMD is a completely non-recursive and adaptive signal processing method recently proposed by Dragomiretskiy. It can determine the frequency center and bandwidth of each component by iteratively searching for the optimal solution of the variational model, so that the effective separation of signal frequency can be adaptively realized. VMD is a variational problem-solving method based on the three concepts of classical Wiener filtering, Hilbert transform, and frequency mixing. To estimate the bandwidth of each modal function $u_{k}$, the following steps construct a variational problem:

(1) Obtaining a corresponding unilateral spectrum by performing a Hilbert transform on each $u_{k}$.
(2) Moving each $u_{k}$ spectrum to a respective estimated center frequency by an exponential hybrid modulation method.
(3) The signal is demodulated according to the Gaussian smoothness and the gradient squared criterion to estimate the bandwidth of each $u_{k}$.

The constrained variational problem can be expressed as:

(1) $$\underset{\left\{u_{k}\right\},\left\{\omega_{k}\right\}}{\min}\left\{{\sum_{k}\left\|\partial_{t}\left[\left(\delta (t)+\frac{j}{\pi t})\cdot u_{k}(t\right)\right]e^{-j\omega_{k}t}\right\|}_{2}^{2}\right\}$$

(2) $$s.t.\sum_{k=1}^{K}u_{k}(t)=f(t)$$

where $u_{k}=\left\{u_{1},u_{2},\cdots u_{K}\right\}$ is the modal function; $\omega_{k}=\left\{\omega_{1},\omega_{2},\cdots \omega_{K}\right\}$ is the center frequency of each modal function. In order to solve the constrained variational problem, the Lagrangian multiplier λ(t) and the quadratic penalty factor α are introduced to transform it into an unconstrained variational problem. The augmented Lagrangian expression is:

(3) $$L(\left\{u_{k}\right\},\left\{\omega_{k}\right\},\lambda )=\alpha {\sum_{k}\left\|\partial_{t}\left[\left(\delta (t)+\frac{j}{\pi t})\cdot u_{k}(t\right)\right]e^{-j\omega_{k}t}\right\|}_{2}^{2}+{\left\|f(t)-\sum_{k}u_{k}(t)\right\|}_{2}^{2}+\langle \lambda (t),f(t)-\sum_{k}u_{k}(t)\rangle$$

Using the multiplication operator alternating direction method, the saddle point of the extended Lagrangian expression is obtained by iteratively updating $u_{k}{}^{k+1}$, $\omega_{k}{}^{k+1}$, $\lambda^{k+1}$. The iteration steps are as follows:

(1) Initialization ${\overset{\wedge }{u}}_{k}^{1}$, $\omega_{k}^{1}$, ${\overset{\wedge }{\lambda }}_{k}^{1}$, n = 0.
(2) The number of iterations n = n + 1.
(3) For *k* = 1:*K*.
  According to formula (4) and formula (5), for all ω≥0, update ${\overset{\wedge }{u}}_{k}$ and $\omega_{k}$,
  (4) $${\overset{\wedge }{u}}_{k}^{n+1}(\omega )=\frac{\overset{\wedge }{f}(\omega )-\sum_{i>k}^{K}{\overset{\wedge }{u}}_{k}^{n+1}(\omega )+\frac{{\overset{\wedge }{\lambda }}^{n}(\omega )}{2}}{1-2\alpha {\left(\omega -\omega_{k}^{n}\right)}^{2}}$$
  (5) $$\omega_{k}^{n+1}=\frac{{\int_{0}^{\infty }\omega \left|{\overset{\wedge }{u}}_{k}^{n+1}(\omega )\right|}^{2}d\omega }{\int_{0}^{\infty }{\left|{\overset{\wedge }{u}}_{k}^{n+1}(\omega )\right|}^{2}d\omega }$$
  where ${\overset{\wedge }{u}}_{k}^{n+1}(\omega ),\;\overset{\wedge }{f}(\omega )$ and ${\overset{\wedge }{\lambda }}_{k}^{n+1}(\omega )$ are the Fourier transforms of their corresponding time domain functions.
(4) According to formula (6), for all ω≥0, double lifting, update λ,
  (6) $${\overset{\wedge }{\lambda }}^{n+1}(\omega )={\overset{\wedge }{\lambda }}^{n}(\omega )+\upsilon \left[\overset{\wedge }{f}(\omega )-\sum_{k=1}^{K}{\overset{\wedge }{u}}_{k}^{n+1}(\omega )\right]$$
  where υ is the noise tolerance and can be set to 0 for good denoising effect.
(5) Repeat steps (2)–(4) until the stop condition $\sum_{k}\left({\left\|{\overset{\wedge }{u}}_{k}^{n+1}-{\overset{\wedge }{u}}_{k}^{n}\right\|}_{2}^{2}/{\left\|{\overset{\wedge }{u}}_{k}^{n}\right\|}_{2}^{2}<\varepsilon \right)$ is satisfied (where ε is the convergence precision and ε > 0), and k modal functions are obtained, and the iterative update ends.

### 2.2. IFOA-VMD Decomposition

#### 2.2.1. Energy Factor

Due to the complexity and variety of the measured signals, the difficulty and key to using the VMD algorithm is how to select the appropriate decomposition number K and the penalty parameter value α [37]. When using VMD, the preset decomposition number K and the penalty parameter α need to be selected. The preset decomposition number K and the penalty parameter α will affect the decomposition result. The smaller the penalty parameter α in the VMD algorithm, the larger the bandwidth of each IMF component obtained by the decomposition; the larger the α, the smaller the bandwidth. If the selected K value is too large or too small, it is easy to cause information loss or redundancy. Based on this, this paper proposes a new method of fusion of FOA and VMD, which can adaptively optimize the internal combination parameters (K,α) of VMD. However, before the parameter optimization, the fitness function needs to be determined. The selection principle of K is that the new central frequency maximum or minimum value no longer appears, thus ensuring that the VMD decomposition does not miss higher or lower center frequencies. Therefore, the IMF component energy growth factor can be used as the fitness function, and the energy growth factor e is defined as the ratio of the energy difference between the current decomposition component and the previous decomposition component to the original signal energy, which can be calculated by equation (7).

(7) $$e=\frac{{\left\|\sum u_{k}-\sum u_{k-1}\right\|}_{2}^{2}}{{\left\|f\right\|}_{2}^{2}}$$

where $\sum u_{k}$ is the energy of the current decomposition component, $\sum u_{k-1}$ is the energy of the last decomposition component, and f is the original signal. In this paper, the energy growth factor is used to determine an indicator of the number of modes, and the energy growth factor threshold e is set to 0.01 [38]. At the same time, the maximum decomposition modulus is set to 10, and the maximum penalty parameter is set to 6000. In the process of decomposing the signal by the VMD, one of the above conditions is satisfied, and the decomposition is stopped. Therefore, the number of decomposition modes and the penalty parameters can be adaptively determined by seeking the minimum value of the energy growth factor, and the parameter optimization process of the VMD is intuitively described as solving the problem of minimization of the fitness function.

#### 2.2.2. FOA-VMD Algorithm

**Figure 1:**
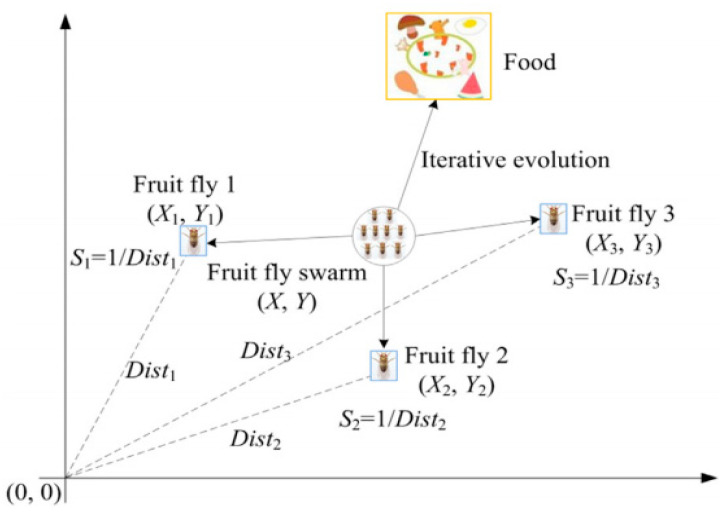
Iterative search for food programs for fruit fly populations.

The fruit fly optimization algorithm (FOA) is a new method based on the global optimization of fruit fly foraging behavior. Drosophila is superior to other species in sensory perception, especially in terms of smell and vision. The olfactory organ can sensitively collect various scents floating in the air and can also find the location of food and companion gathering through clear vision and then fly in this direction. Therefore, the fruit fly algorithm can be applied to solve the optimal problem [18]. Figure 1 shows a schematic representation of iterative search for food in Drosophila populations. The main process of the FOA-VMD algorithm is as follows:

The first step: first assign the population size sizepop, the maximum number of iterations maxgen. Let sizepop = 10, maxgen = 20.

The second step: initialize the initial position coordinates of the fruit fly population and initialize the initial position of the individual flies according to the range of variation of the parameters. Wherein the decomposition number K ranges from 1 to 10, and the penalty parameter α ranges from 200 to 6000. Init X_axis, Init Y_axis.

The third step: according to the behavior of fruit flies searching for food, give the fruit flies a random direction and distance for foraging. The choice of random distance is determined according to the initial coordinates, generally two orders of magnitude.

(8) $$\{\begin{array}{c} \mathrm{Xi}=X\_\mathrm{axis}+\mathrm{Random}\;\mathrm{Value}\\ \mathrm{Yi}=Y\_\mathrm{axis}+\mathrm{Random}\;\mathrm{Value} \end{array}$$

The fourth step: Since the position of the food cannot be known, first estimate the distance from the origin (Dist) and then calculate the taste concentration determination value (Si), which is the reciprocal of the distance.

(9) $$\{\begin{array}{c} {\mathrm{Disti}=\mathrm{sqrt}(\mathrm{Xi}}^{2}{+\mathrm{Yi}}^{2})\\ \mathrm{Si}=1/\mathrm{Disti}\; \end{array}$$

The fifth step: the taste concentration determination value (Si) is substituted into a taste density determination function (or a fitness function) to determine the taste concentration (Smell(i)) of the individual position of the fruit fly. The energy growth factor e is used as the concentration determination function, that is, the energy growth rate minimum value is calculated, and the lower threshold value of e is set to 0.01.

(10) $$\mathrm{Smell}(i)=\mathrm{Function}(\mathrm{Si})\;\mathrm{Function}(\mathrm{Si})=e=\frac{{\left\|\sum u_{k}-\sum u_{k-1}\right\|}_{2}^{2}}{{\left\|f\right\|}_{2}^{2}}$$

The sixth step: Bring the taste concentration determination value into the concentration determination function to determine the taste concentration value of the fruit fly individual, find the fruit fly with the lowest taste concentration in the fruit fly group, and obtain the minimum value of the energy growth factor e.

(11) [bestSmell bestIndex]=min(Smell)

The seventh step: Preserve the optimal taste concentration value and the coordinates of x and y. At this time, the fruit fly group uses vision to fly to the position and record the values of the best parameters *K* and α.

(12) $$\{\begin{array}{c} \mathrm{Smellbest}=\mathrm{bestSmell}\\ X\_\mathrm{axis}=X(\mathrm{bestIndex})\\ Y\_\mathrm{axis}=Y(\mathrm{bestIndex}) \end{array}$$

The eighth step: Enter iterative search, repeat steps 2–5, and determine whether the taste concentration is better than the previous iteration taste concentration. If yes, proceed to step 6, until the number of runs meets maxgen times, stop the operation, and output the best taste concentration value and the best parameters.

#### 2.2.3. Improved FOA Algorithm

**Figure 2:**
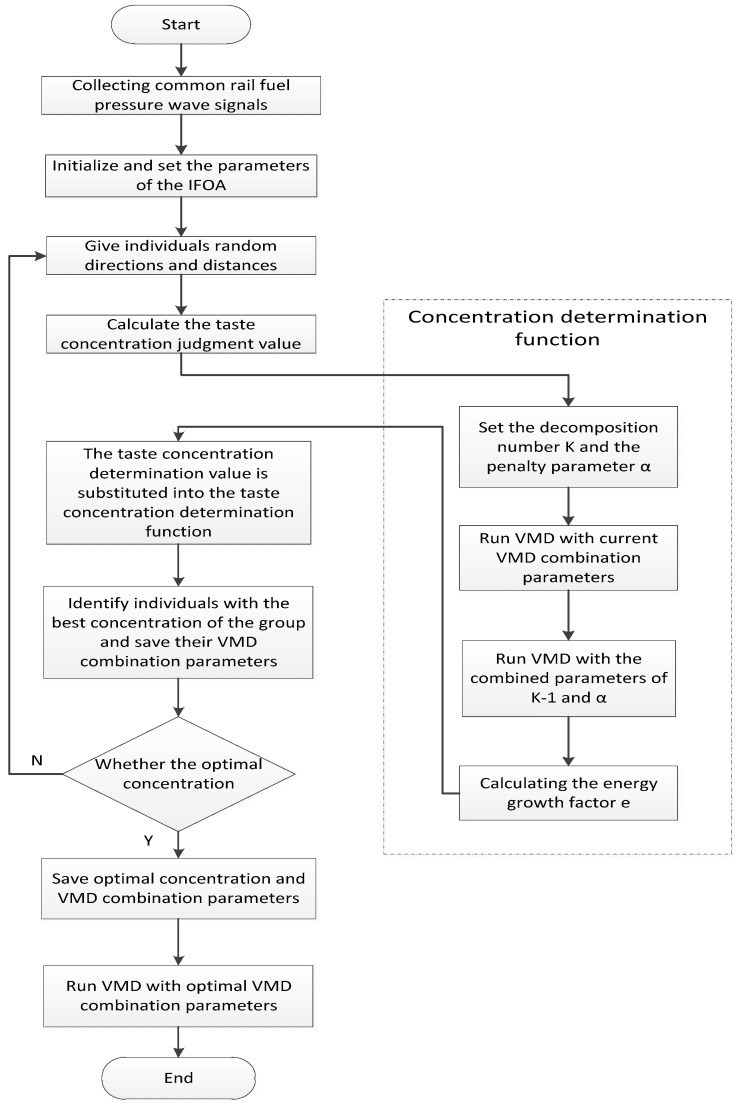
IFOA-VMD algorithm flow chart.

In order to avoid the FOA falling into local optimization and improve the convergence speed and convergence precision of the algorithm, this paper keeps the process of learning the lessons learned from the worst individuals while retaining the FOA algorithm to the optimal individual learning process, that is, increasing the “reverse cognition” part. The improvement strategy is to adjust Equations (11) and (12) to Equations (13) and (14), respectively. At the same time, the degree of learning from the optimal and worst individuals can also be adjusted by the linearly decreasing dynamic coefficient ω. At the beginning of the iteration, ω is larger, mainly to learn from the optimal individual and improve the convergence speed of the algorithm; after continuous iteration, ω gradually decreases, at which time the learning to the worst individual is enhanced, and the learning to the optimal individual is weakened so that the algorithm has the ability to jump out of the local extremum. At the same time, in the case that ω does not decrement to 0, the individual Drosophila can always learn from the optimal individual, thus improving the convergence speed and convergence precision of the algorithm. The flow chart for optimizing the VMD parameters using IFOA is shown in Figure 2.

(13) $$\{\begin{array}{c} \left[\mathrm{bestSmell}\;\mathrm{bestIndex}]=\min(\mathrm{Smell}\right)\\ \left[\mathrm{worstSmell}\;\mathrm{worstIndex}]=\max(\mathrm{Smell}\right) \end{array}$$

(14) $$\{\begin{array}{c} \mathrm{Smellbest}=\mathrm{bestSmell},\mathrm{Smellworst}=\mathrm{worstSmell}\\ X(\mathrm{best})=X(\mathrm{bestIndex}),Y(\mathrm{best})=Y(\mathrm{bestIndex})\\ X(\mathrm{worst})=X(\mathrm{worstIndex}),Y(\mathrm{worst})=Y(\mathrm{worstIndex})\\ X\_\mathrm{axis}=\omega X(\mathrm{bestIndex})‒(1‒\omega )X(\mathrm{worstIndex}),Y\_\mathrm{axis}=\omega Y(\mathrm{bestIndex})‒(1‒\omega )Y(\mathrm{worstIndex}) \end{array}$$

**Figure 3:**
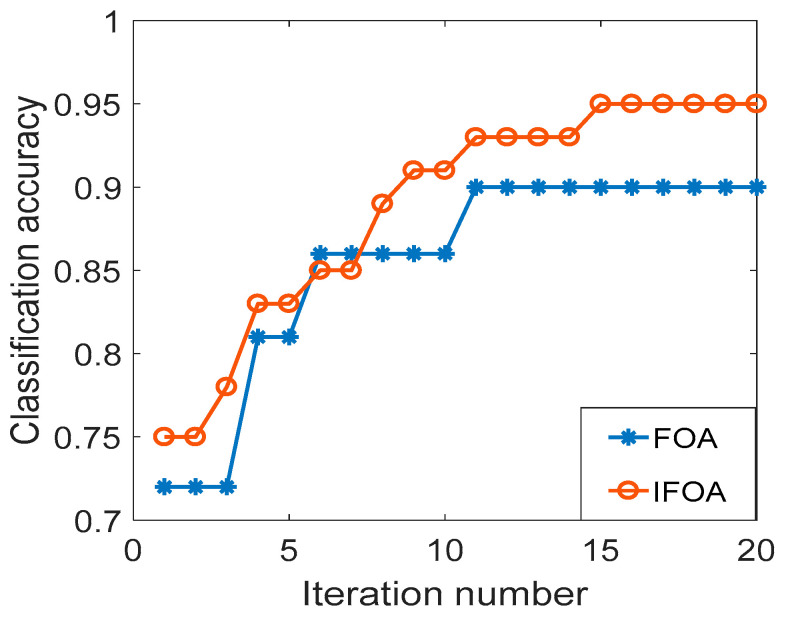
Wine data set classification accuracy curve.

In order to verify that the IFOA algorithm can reduce the local optimization probability and improve the convergence accuracy, the Wine dataset in the UCI database is used to compare the performance of IFOA and FOA. The feature dimension is 13, categories is 3, and the training sample and test sample are respectively 89. The data set is input into the SVM for classification and identification. Figure 3 is the optimization iteration curve of the cross-validation classification accuracy of the Wine data set, where Sizepop is 10 and Maxgen is 20. As can be seen from Figure 2, the FOA curve is relatively monotonous, and it is difficult to jump out of the local optimum. After classification calculation, the classification accuracy of IFOA is 97.75%, the algorithm calculation time is 14.25 s, the classification accuracy of FOA is 92.69%, the calculation time of the algorithm is 12.98 s, and the classification accuracy of IFOA is increased by 5.06%, indicating that IFOA has higher convergence precision. Since IFOA has increased the information on the worst individual information and location update of the population, the calculation time is slightly longer than FOA, but the difference between the two is not large.

## 3. Hierarchical Dispersion Entropy

The IFOA-VMD method can obtain the IMF component sensitive to the injector fault characteristics, but how to accurately extract the fault state characteristic information from the nonlinear fuel pressure wave signal is an urgent problem to be solved. Comprehensive and accurate reflection of fault characteristics information is the premise of high-precision fault diagnosis. Therefore, this paper proposes a method to extract the hierarchical dispersion entropy (HDE) as the fault feature, which can reflect the fault characteristics of the injector as a whole, providing more comprehensive and accurate time mode information.

### 3.1. Hierarchical Dispersion Entropy Algorithm Flow

Referring to the advantages of hierarchical segmentation in hierarchical entropy, combined with the definition of dispersion entropy, the concept of hierarchical dispersion entropy is proposed. The calculation process of hierarchical dispersion entropy is as follows:

(i) Given a time series {u(i),i=1,2,…,N} with the length *N*($N=2^{n}$, *n* is a positive integer), define the averaging operators $Q_{0}$ and $Q_{1}$ for the time series as follows:

(15) $$Q_{0}(u)=\frac{u(2j)+u(2j+1)}{2},j=1,2,\ldots ,2^{n-1}$$

(16) $$Q_{1}(u)=\frac{u(2j)-u(2j+1)}{2},j=1,2,\ldots ,2^{n-1}$$

where $Q_{0}(u)$ and $Q_{1}(u)$ carry the low-frequency and high-frequency features of u at scale 2, respectively.

When j = 0 or j = 1, the matrix operator $Q_{j}$ is defined as follows

(17) $$Q_{j}={\left[\begin{array}{cccc} \frac{1}{2} & \frac{(-1{)}^{j}}{2} & 0 & 0\\ 0 & 0 & \frac{1}{2} & \frac{(-1{)}^{j}}{2}\\ \vdots & \vdots & \vdots & \vdots \\ 0 & 0 & 0 & 0 \end{array}\begin{array}{cccc} \ldots & & 0 & 0\\ \ldots & & \vdots & \vdots \\ \ldots & & 0 & 0\\ \ldots & & \frac{1}{2} & \frac{(-1{)}^{j}}{2} \end{array}\right]}_{2^{n-1}\times 2^{n}}$$

(ii) In order to perform the hierarchical analysis on the signal *u*(*i*), the above operators have to be employed iteratively. Let k∈N construct a vector $\left[\gamma_{1,}\gamma_{2,}\ldots ,\gamma_{k}\right]\in \left\{0,1\right\}$, then the integer e can be expressed as

(18) $$e=\sum_{j=1}^{k}\gamma_{j}2^{k-j}$$

**Figure 4:**
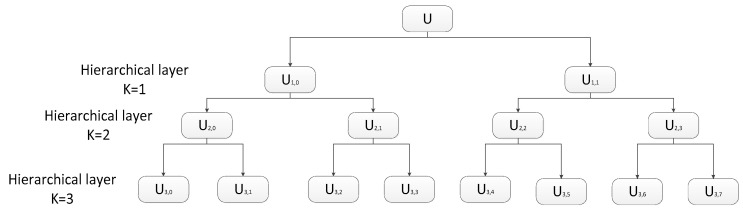
Hierarchical decomposition with three layers.

In the formula, the vector corresponding to the positive integer e is $\left[\gamma_{1,}\gamma_{2,}\ldots ,\gamma_{n}\right]$; *k* and *e* are the layer number and node number, respectively.

(iii) Based on the vector $\left[\gamma_{1,}\gamma_{2,}\ldots ,\gamma_{n}\right]$, the hierarchical component $u_{k,e}$ is expressed as

(19) $$u_{k,e}=Q_{\gamma_{1}},Q_{\gamma_{2}},\ldots ,Q_{\gamma_{k}}(u)$$

where *k* represents the *k*-layer in the hierarchical segmentation, and the original time series *u*(*i*) is represented by $u_{k,0}$ and $u_{k,1}$ in the low-frequency portion and the high-frequency portion of the k + 1 layer. For different *k* and *e*, the signals $u_{k,e}$ consist of the hierarchical decomposition of signal *u*(*i*) in different scales. In Figure 4, the hierarchical decomposition of *u*(*i*) in 4 scales is illustrated in the form of a hierarchical tree.

(iv) Find the dispersion entropy of each hierarchical component obtained and obtain the dispersion entropy of $2^{k}$ hierarchical component [31]. The hierarchical component sequence $u_{k,e}$ is mapped to $\left[y_{1},y_{2},\ldots ,y_{2^{k}}\right]$ by introducing a normal cumulative distribution (NCDF). The calculation formula is $y_{2^{k}}=\frac{1}{\sigma \sqrt{2\pi }}\int_{-\infty }^{u_{k,e}}e^{\frac{-{\left(t-u\right)}^{2}}{2\sigma^{2}}}dt$, and the value ranges from 0 to 1. Next, we assign each y to an integer class with labels from 1 to *c*. For each member of the mapped signal, we use $z_{j}^{c}=round(c*y_{j}+0.5)$, where $z_{j}^{c}$ shows the jth member of the classification time series.

(v) Introducing the embedded dimension m and the delay parameter *d* and reconstructing the sequence, $z_{i}^{m,c}$ is

(20) $$z_{i}^{m,c}=\left\{z_{i}^{c},z_{i+d}^{c},\ldots ,z_{i+(m-1)d}^{c}\right\},i=1,2,\ldots ,N-(m-1)d$$

Each time series $z_{i}^{m,c}$ is mapped to a dispersion pattern $\pi_{v_{0}v_{1}\ldots v_{m-1}}$, where $z_{i}^{c}=v_{0},z_{i+d}^{c}=v_{1},\ldots ,z_{i+(m-1)d}^{c}=v_{m-1}$. The number of dispersion patterns that can be assigned to each time series $z_{i}^{m,c}$ is equal to $c^{m}$ since the signal has m members and each member can be one of the integers from 1 to *c*.

(vi) For each of $c^{m}$ dispersion pattern, relative frequency is obtained as follows:

(21) $$p(\pi_{v_{0}\ldots v_{m-1}})=\frac{Number\left\{i|i\leq N-(m-1)d,z_{i}^{m,c}has\underset{}{}type\pi_{v_{0}\ldots v_{m-1}}\right\}}{N-(m-1)d}$$

In fact, $p(\pi_{v_{0}\ldots v_{m-1}})$ shows that the number of dispersion patterns $\pi_{v_{0}v_{1}\ldots v_{m-1}}$ that are assigned to $z_{i}^{m,c}$ divided by the total number of embedding signals with embedding dimension *m*.

(vii) Based on the definition of information entropy, the single dispersion entropy is

(22) $$e(u,m,c,d)=-\sum_{\pi =1}^{c^{m}}p(\pi_{v_{0}\ldots v_{m-1}})\cdot \ln(p(\pi_{v_{0}\ldots v_{m-1}}))$$

Hierarchical dispersion entropy can be expressed as

(23) $$HDE=E(u_{k,e},m,c,d)$$

### 3.2. Parameter Selection

According to the definition of hierarchical dispersion entropy, four parameters need to be set before the calculation of hierarchical fuzzy entropy: signal length *N*, embedding dimension m, class number *c*, and decomposition layer number *k*. Since the *k* value is too large, it affects the computational efficiency and causes the points involved in each hierarchical component calculation to decrease. At the same time, if the k value is too small, the original sequence band division is not detailed enough to obtain sufficient gradation components from low frequency to high frequency. This paper sets the number of decomposition layers *k* = 3. In order to evaluate the sensitivity of hierarchical dispersion entropy to signal length *N*, embedding dimension m and class number, 40 sets of hierarchical dispersion entropy of white noise and 1/*f* noise of different lengths are calculated, and 40 different levels are calculated. The mean and standard deviation of the nodes are determined by the coefficient of variation (CV), where the coefficient of variation is CV = standard deviation/mean.

**Figure 5:**
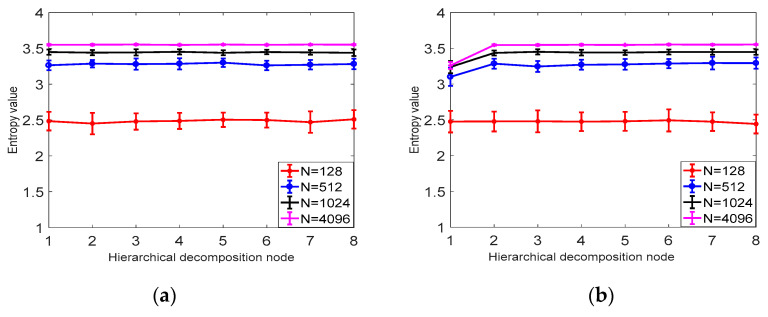
(**a**) The mean and SD results of HDE for 40 sets of white noise time series of different lengths. (**b**) Mean and SD results of 40 sets of HDE with different length 1/*f* noise time series.

**Figure 6:**
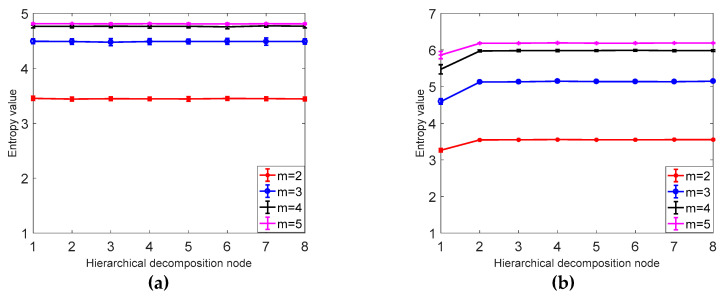
(**a**) The average and SD results of HDE for 40 different sets of embedded white noise time series. (**b**) The mean and SD results of 40 sets of HDE with different embedded dimension 1/*f* noise time series.

**Figure 7:**
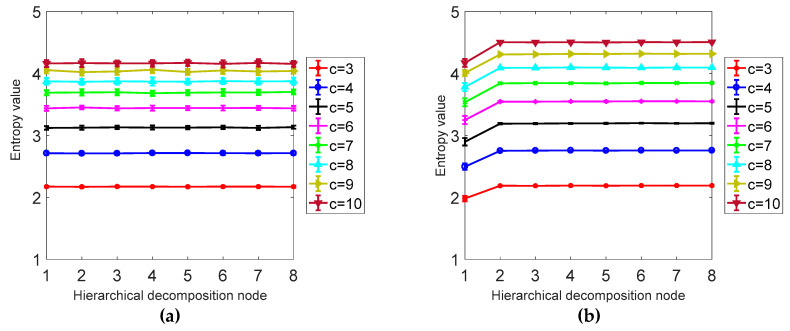
(**a**) Mean and SD results of HDE for 40 different white noise time series. (**b**) The mean and SD results of HDE for 40 different classes of 1/*f* noise time series.

As shown in Figure 5, it can be concluded from a and b that the larger the signal length *N* is, the higher the stability is, and the smaller the error bar is, the difference between *N* = 1024 and *N* = 4096 is not obvious; from Table 1 it can be seen that as the signal length is larger, the smaller the CV value is, the more stable the calculation of HDE is. In this paper, *N* = 1024 is selected as the optimal signal length. It can be seen from Table 2 and Figure 6 that the CV value of the embedding dimension *m* = 2 is small, indicating that the HDE value of *m* = 2 is high and the error is small. In this paper, *m* = 2 is selected as the optimal embedding dimension. It can be seen from the CV values of different classes in Table 3 and Figure 7. As *C* increases, the CV value increases, the coefficient of variation of *c* = 3 is the smallest, and the error rate is the lowest. *C* = 3 is the best class.

### 3.3. Comparison with Other Methods

**Figure 8:**
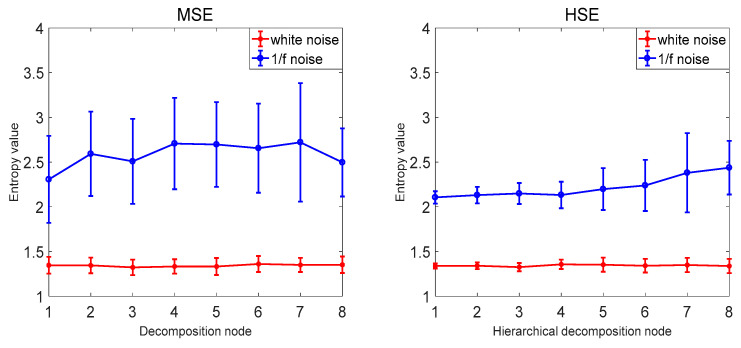

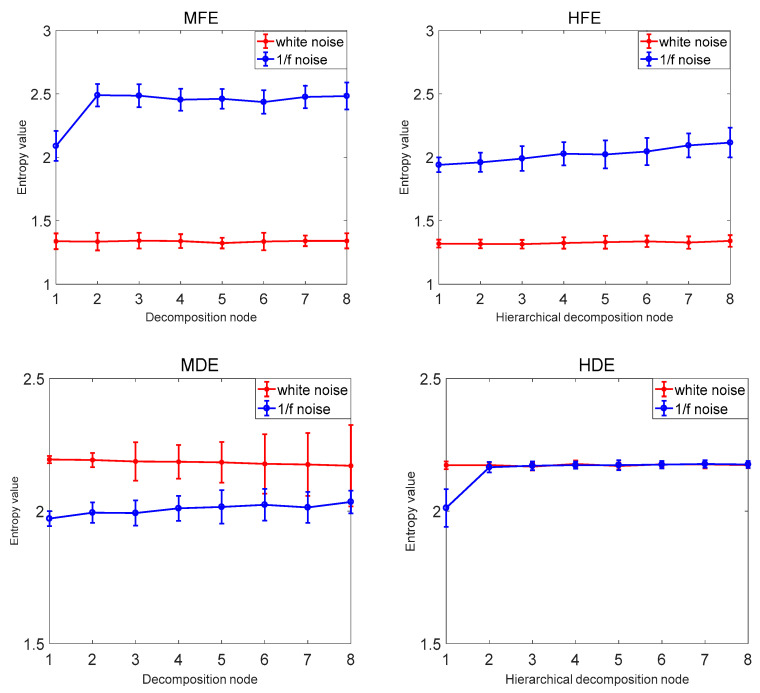
Average and SD results of MSE, HSE, MFE, HFE, MDE, and HDE for 40 white noise and 1/*f* noise time series.

In order to verify that the proposed hierarchical dispersion entropy method is better than the current information entropy method, this paper compares the hierarchical dispersion entropy with multi-scale sample entropy, hierarchical sample entropy, multi-scale fuzzy entropy, hierarchical fuzzy entropy, and multi-scale dispersion entropy. A total of 40 sets of white noise and 1/*f* noise were used as information entropy to calculate the samples, and the coefficient of variation CV of the same decomposition node of each information entropy was compared. The information entropy parameter selection is as follows: the decomposition layer number *k* = 3, the signal length *N* = 1024, the class number *c* = 3, the embedding dimension *m* = 2, the scale factor τ=8. The comparison results are shown in Figure 8 and Table 4. The MSE and HSE are compared as examples. As the decomposition scale increases, the stability of HSE is significantly higher than that of MSE, and the CV value of HSE of decomposition node 4 is also smaller than MSE, indicating that the hierarchical entropy performance is significantly better than multi-scale entropy; the hierarchical entropy can consider the high and low frequency components, and extract the time mode information more comprehensively and accurately; taking MDE, MSE and MFE as examples to compare, with the increase of the decomposition scale, MDE is more stable than MSE and MFE. The CV value of the decomposition node 4 is also the smallest MDE and the error bar is the smallest, indicating that the performance of the dispersion entropy is significantly better than the sample entropy and the fuzzy entropy. The anti-noise of the dispersion entropy is better, and the bandwidth variation is more sensitive, and it is able to map status information more accurately. It shows that the CV value of HDE is the smallest and the calculation is the most stable. The HDE method is better than the existing public information entropy method, which not only improves the stability of entropy calculation but also reduces the bit error rate of entropy calculation.

## 4. Engineering Test Verification

### 4.1. Signal Acquisition

**Figure 9:**
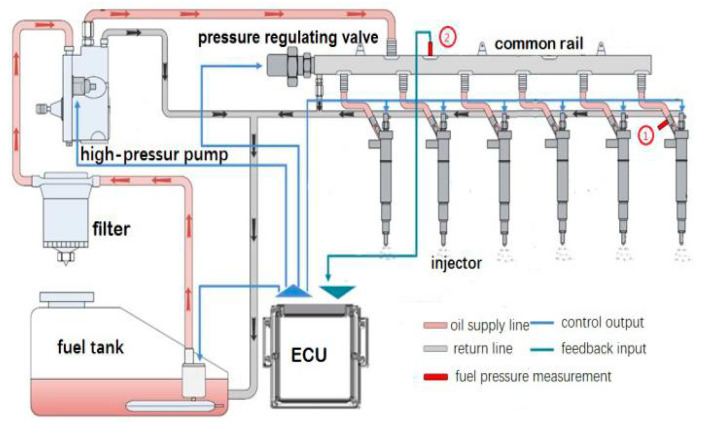
High-pressure common rail fuel injection system.

In order to verify the effectiveness of the proposed fault diagnosis algorithm, the experimental data comes from the high-pressure common rail system established by the School of Power Engineering of Harbin Engineering University, as shown in Figure 9. The system includes a fuel tank, a high-pressure oil pump, a pressure regulating valve, a common rail and a fuel injector.

**Figure 10:**
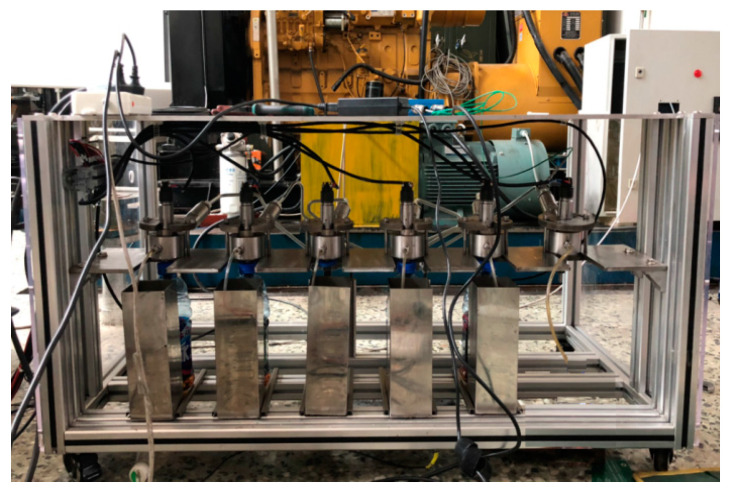
High-pressure common rail test system.

The picture of real high-pressure common rail experiment system is shown in Figure 10. The structure of this experiment system is the same as the real fuel injection system of 6K420LN-C31 diesel engine, which is made by China Yuchai factory. In this system, there are 6 injectors Bosch crin2 in total. The high-pressure pump is driven by an electric motor. The rail pressure close loop control and fuel injecting in turn are realized by ECU.

In order to study the fault diagnosis method, different working states were simulated on the experimental system. Under the conditions of rail pressure 80 Mpa and injection pulse width 1.5 ms, the injector fault is simulated in No. 1 injector, including nozzle clogging and solenoid valve failure. The solenoid valve failure state is that the solenoid valve is stagnant, and the needle valve cannot be seated. The nozzle clogging fault condition is blocked by half of the injector nozzle diameter. The rail pressure was tested at the position 2 of the common rail pressure sensor of Figure 9, and the common rail pressure of the injector in normal operation, solenoid valve failure, and nozzle clogging were obtained, and the sampling frequency was 5 kHz.

### 4.2. Analysis of Test Data

In order to verify the effectiveness of the fusion algorithm of IFOA-VMD and hierarchical dispersion entropy (HDE) in fuel injector fault feature extraction, it is applied to the test data analysis of high-pressure common rail injector.

**Figure 11:**
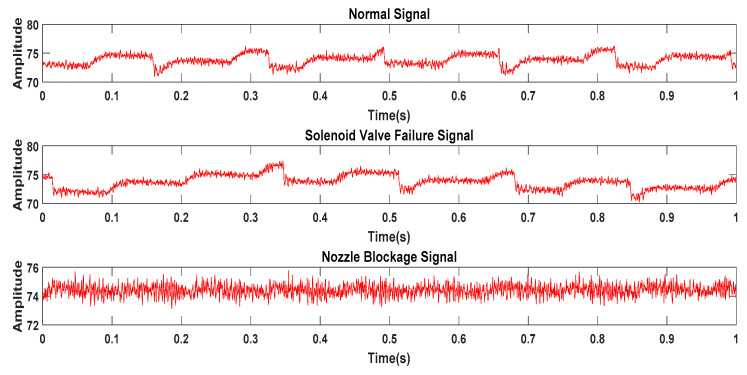
Pressure wave original signal in three states of the injector.

First, on the high-pressure common rail experimental platform, the pressure wave signals of the injector under three working conditions are obtained, including normal operation, solenoid valve failure, and nozzle clogging. The data in a working cycle of diesel engine is show in Figure 11.

**Figure 12:**
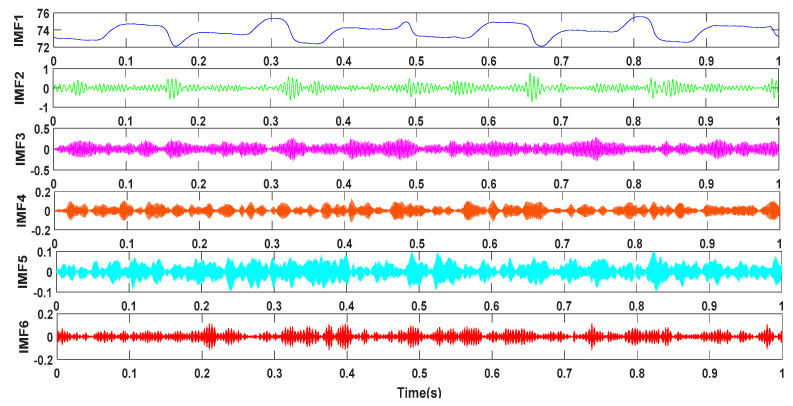
IF0A-VMD decomposition results under normal conditions.

Then, the collected fuel pressure wave signal is decomposed, and the pressure wave signals of the three working states input into the IFOA-VMD algorithm. Taking the normal working state as an example, the IFOA algorithm sets sizepop = 10, maxgen = 20, and the VMD algorithm sets the center frequency init = 1 to terminate the condition $tol=1\times 10^{-7}$. The energy growth factor is used as the concentration determination function, and the optimal *K* = 6, α = 6000 is obtained by the IFOA algorithm. Next, the pressure wave signal is run with the optimal *K* and the combined parameter α to obtain the IMF component, and the results are shown in Figure 12.

Then, the state characteristic parameters of the IMF components of the three working state pressure wave signals decomposed by the IFOA-VMD are extracted, and the hierarchical dispersion entropy of the fuel pressure wave IMF component obtained by the IFOA-VMD algorithm is calculated. In this paper, *m* = 2, *c* = 3, *k* = 3, and *N* = 1024 are set. The hierarchical dispersion entropy of the IMF components of the three working states is calculated, and the feature extraction of the fuel pressure wave signals of the three working states is completed.

Finally, the hierarchical dispersion entropy of IMF components in different working conditions is regarded as the feature vector for fault state classification and recognition, and the input support vector machine is used for classification and recognition. After obtaining the HDE characteristic parameters, it is necessary to establish an intelligent classifier to realize the automatic fault diagnosis of the injector. When dealing with multi-class classification problems, it is necessary to construct a suitable multi-class classifier. Currently, indirect methods are mainly used to construct multi-class SVM classifiers, such as one-to-one (OVR), one-to-one (OVO), directed acyclic graphs (DAG), partial binary trees (PBT), etc. [39]. This paper uses PBT to construct a multi-fault classifier. The kernel function is an important part of SVM, including linear kernel function, polynomial kernel function, RBF kernel function, and Sigmoid kernel function. The RBF kernel function can approximate nonlinear functions with faster convergence speed and better generalization ability. Therefore, this paper uses the RBF kernel function for classification, with a penalty factor of *C* = 1000.

**Figure 13:**
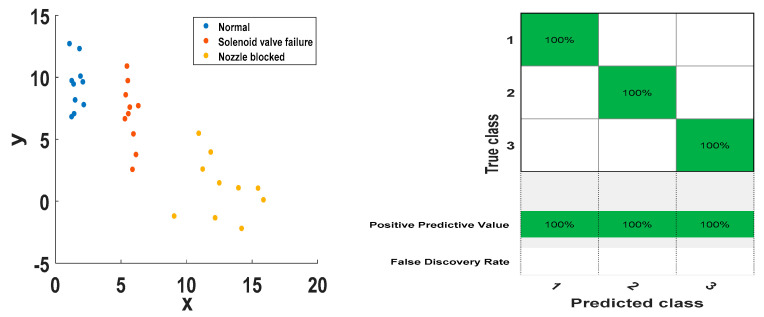
Training sample SVM classification results.

**Figure 14:**
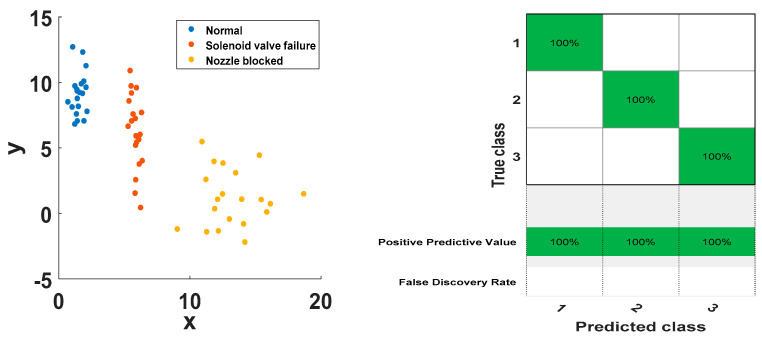
Test sample SVM classification results.

Since there are three categories of injector status classification problems, two SVMs (SVM1 and SVM2) need to be built. The 10 sets of feature vectors of the three states are input into the SVM classifier as the training samples, and the 20 sets of feature vectors are used as the test samples of the input SVM classifier. The classification results are shown in Figure 13 and Figure 14. The classification results of the support vector machine for all training samples and test samples are shown in the Figure 13 and Figure 14. It can be seen that there are obvious boundaries between the three working state feature vectors of the injector, and the prediction classification of the training set and the test set are consistent with the actual classification, and the classification correct rate is 100%. The results show that the method has better classification effect and has better applicability and effectiveness in fault diagnosis and state detection of high-pressure common rail injectors.

### 4.3. Comparative Study of Diagnostic Methods

**Figure 15:**
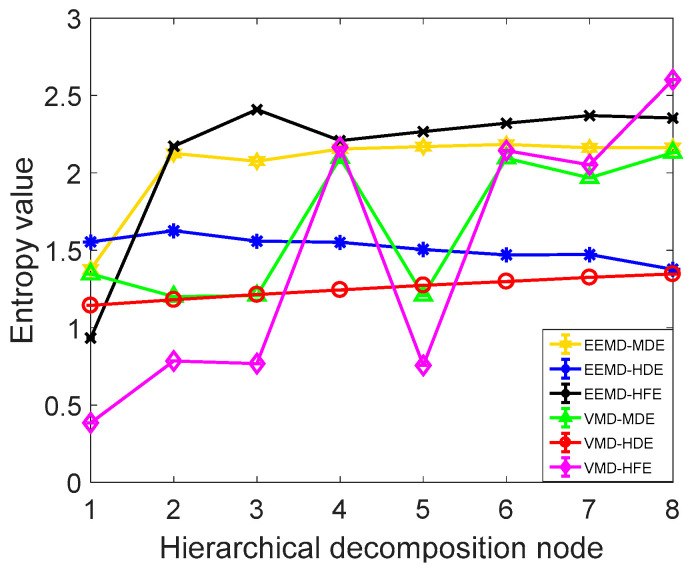
Information entropy characteristics of the IMF1 component.

In order to further verify the superiority of the proposed method, it is compared with the commonly used signal decomposition method and the commonly used information entropy method. Using EEMD-MDE, EEMD-HFE, EEMD-HDE, VMD-HFE, VMD-MDE, and VMD-HDE methods to extract the fuel jet pressure wave signal fault characteristics, taking the normal operating condition fuel pressure wave IMF1 component as an example, the extracted information entropy characteristics are shown in Figure 15. As shown in Figure 15 and Table 5, the CV value and calculation time of the VMD-HDE are the smallest, which indicates that the method has the best performance in evaluating the time series complexity, and it has high calculation accuracy, good stability, and the highest computational efficiency.

Then, the decomposed IMF component is classified and identified as the feature vector input SVM. The 10 sets of feature vectors of the three states are input into the SVM classifier as the training samples, and the 20 sets of feature vectors are used as the test samples of the input SVM classifier. The classification results are shown in Table 6. Taking EEMD-HFE and VMD-HFE, EEMD-MDE and VMD-MDE, EEMD-HDE and VMD-HDE as examples, the classification accuracy of VMD is higher than EEMD, and the calculation time is short. The computational efficiency is high. Taking VMD-HFE, VMD-MDE, and VMD-HDE as examples, HDE performs better on time series complexity metrics and has the highest computational efficiency. Therefore, the effectiveness and superiority of the proposed high-voltage common rail injector fault diagnosis method based on IFOA-VMD and hierarchical dispersion entropy are verified.

## 5. Conclusions

High-precision fault diagnosis of high-pressure common rail injectors is an important means to ensure the safe operation of high-pressure common rail diesel engines. Firstly, this paper utilizes the advantages of high efficiency and robustness of VMD to adaptively decompose fuel pressure waves. However, in order to overcome the shortcomings of VMD’s manual parameter selection, this paper proposes an improved method of IFOA-VMD, which uses minimum energy growth. The factor e is used to optimize the VMD internal combining parameters *K* and α, and the plurality of IMF component signals can be adaptively separated. Then, the hierarchical dispersion entropy is developed to extract the fuel pressure wave fault characteristics, and compared with the existing common information entropy, it is verified that the hierarchical dispersion entropy can fully and accurately reflect the fuel pressure wave signal complexity and that the performance is optimal. Finally, a fault diagnosis algorithm for high-pressure common rail injector based on IFOA-VMD and hierarchical dispersion entropy is proposed. The algorithm integrates the advantages of IFOA-VMD and HDE, which can not only effectively decompose multi-component signals but also suppress the decomposition error of VMD. Moreover, the accuracy of the injector fault diagnosis is improved and extensively compared with various fault diagnosis methods. Based on numerical examples, this method is superior to EEMD from the perspective of calculation time and classification accuracy. Finally, we plan to compare it to other methods (such as fast iterative filtering [15]) in future work.

## Figures and Tables

**Table 1:** CV values of node 4 of hierarchical decomposition of different signal lengths.

Signal Length *N*	128	512	1024	4096
White noise	0.0446	0.0237	0.0105	0.0022
1/*f* noise	0.0527	0.021	0.0114	0.0026

**Table 2:** CV values of node 4 of hierarchical decomposition of different embedding dimensions.

Embedding Dimension *m*	2	3	4	5
White noise	0.0028	0.0118	0.0065	0.0026
1/*f* noise	0.0022	0.0055	0.0052	0.0023

**Table 3:** CV values of node 4 of hierarchical decomposition of different classes.

Class *c*	3	4	5	6	7	8	9	10
White noise	0.0053	0.0082	0.0105	0.0115	0.0115	0.0168	0.0128	0.0123
1/*f* noise	0.0014	0.0022	0.0024	0.0024	0.0021	0.0029	0.0029	0.0043

**Table 4:** CV values of decomposition node 4 with different information entropy.

Information Entropy Method	MSE	HSE	MFE	HFE	MDE	HDE
White noise	0.0601	0.0385	0.0406	0.0338	0.0291	0.0061
1/*f* noise	0.1885	0.0696	0.0455	0.0354	0.0233	0.0062

**Table 5:** CV value and calculation time of feature extraction method.

Method	EEMD-HFE	EEMD-MDE	EEMD-HDE	VMD-HFE	VMD-MDE	VMD-HDE
CV/10^−16^	4.72	3.87	2.57	4.52	2.88	2.03
Time/s	17.858	2.932	1.985	16.985	2.287	1.941

**Table 6:** Comparison of classification results of feature extraction methods.

Method	EEMD-HFE	EEMD-MDE	EEMD-HDE	VMD-HFE	VMD-MDE	VMD-HDE
Classification accuracy/%	88.8	92.2	93.3	95.5	97.7	100
Time/s	20.8	11.9	11.4	13.7	4.7	4.2

## References

[1] Yang Y.-S., Ming A.-B., Zhang Y.-Y., Zhu Y.-S. (2017). Discriminative non-negative matrix factorization (DNMF) and its application to the fault diagnosis of diesel engine. Mech. Syst. Sig. Process..

[2] Kowalski J., Krawczyk B., Woźniak M. (2017). Fault diagnosis of marine 4-stroke diesel engines using a one-vs-one extreme learning ensemble. Eng. Appl. Artif. Intell..

[3] Gao Z., Ma C., Song D., Liu Y. (2017). Deep quantum inspired neural network with application to aircraft fuel system fault diagnosis. Neurocomputing.

[4] Taghizadeh-Alisaraei A., Mahdavian A. (2019). Fault detection of injectors in diesel engines using vibration time-frequency analysis. Appl. Acoust..

[5] Lee Y., Lee C.H. (2018). An uncertainty analysis of the time-resolved fuel injection pressure wave based on BOSCH method for a common rail diesel injector with a varying current wave pattern. J. Mech. Sci. Technol..

[6] Yang Y., Peng Z., Zhang W., Meng G. (2019). Parameterised time-frequency analysis methods and their engineering applications: A review of recent advances. Mech. Syst. Sig. Process..

[7] Li L., Cai H., Jiang Q., Ji H. (2019). An empirical signal separation algorithm for multicomponent signals based on linear time-frequency analysis. Mech. Syst. Sig. Process..

[8] Sun R., Yang Z., Chen X., Tian S., Xie Y. (2018). Gear fault diagnosis based on the structured sparsity time-frequency analysis. Mech. Syst. Sig. Process..

[9] Wu Y., Li X., Wang Y. (2018). Extraction and classification of acoustic scattering from underwater target based on Wigner-Ville distribution. Appl. Acoust..

[10] Qu H., Li T., Chen G. (2019). Synchro-squeezed adaptive wavelet transform with optimum parameters for arbitrary time series. Mech. Syst. Sig. Process..

[11] Mohanty S., Gupta K.K., Raju K.S. (2018). Hurst based vibro-acoustic feature extraction of bearing using EMD and VMD. Measurement.

[12] Lin L., Wang Y., Zhou H. (2009). Iterative filtering as an alternative algorithm for empirical mode decomposition. Adv. Adapt. Data Anal..

[13] Cicone A., Liu J., Zhou H. (2016). Adaptive local iterative filtering for signal decomposition and instantaneous frequency analysis. Appl. Comput. Harmon. Anal..

[14] Cicone A. (2019). Nonstationary signal decomposition for dummies. Advances in Mathematical Methods and High Performance Computing.

[15] Cicone A., Zhou H. (2018). Numerical Analysis for Iterative Filtering with New Efficient Implementations Based on FFT. arXiv Preprint.

[16] Wang L., Liu Z., Miao Q., Zhang X. (2018). Time–frequency analysis based on ensemble local mean decomposition and fast kurtogram for rotating machinery fault diagnosis. Mech. Syst. Sig. Process..

[17] Dragomiretskiy K., Zosso D. (2013). Variational mode decomposition. IEEE Trans. Signal Process..

[18] Bi F., Li X., Liu C., Tian C., Ma T., Yang X. (2019). Knock detection based on the optimized variational mode decomposition. Measurement.

[19] Chen X., Yang Y., Cui Z., Shen J. (2019). Vibration fault diagnosis of wind turbines based on variational mode decomposition and energy entropy. Energy.

[20] Li G., Tang G., Luo G., Wang H. (2019). Underdetermined blind separation of bearing faults in hyperplane space with variational mode decomposition. Mech. Syst. Sig. Process..

[21] Wu L., Yang Y., Maheshwari M., Li N. (2019). Parameter optimization for FPSO design using an improved FOA and IFOA-BP neural network. Ocean Eng..

[22] Wang L., Lv S.-X., Zeng Y.-R. (2018). Effective sparse adaboost method with ESN and FOA for industrial electricity consumption forecasting in China. Energy.

[23] Cheng J., Xiong Y. (2017). The quality evaluation of classroom teaching based on FOA-GRNN. Procedia Comput. Sci..

[24] Han S.-Z., Huang L.-H., Zhou Y.-Y., Liu Z.-L. (2018). Mixed chaotic FOA with GRNN to construction of a mutual fund forecasting model. Cognit. Syst. Res..

[25] Pincus S. (1995). Approximate entropy (ApEn) as a complexity measure. Chaos: An Interdisciplinary Journal of Nonlinear Science.

[26] Richman J.S., Moorman J.R. (2000). Physiological time-series analysis using approximate entropy and sample entropy. Am. J. Physiol. Heart Circ. Physiol..

[27] Costa M., Goldberger A.L., Peng C.-K. (2005). Multiscale entropy analysis of biological signals. Phys. Rev. E.

[28] Costa M., Goldberger A.L., Peng C.-K. (2002). Multiscale entropy analysis of complex physiologic time series. Phys. Rev. Lett..

[29] Zheng J., Pan H., Cheng J. (2017). Rolling bearing fault detection and diagnosis based on composite multiscale fuzzy entropy and ensemble support vector machines. Mech. Syst. Sig. Process..

[30] Jiang Y., Peng C.-K., Xu Y. (2011). Hierarchical entropy analysis for biological signals. J. Comput. Appl. Math..

[31] Rostaghi M., Azami H. (2016). Dispersion entropy: A measure for time-series analysis. IEEE Signal Process Lett..

[32] Azami H., Arnold S.E., Sanei S., Chang Z., Sapiro G., Escudero J., Gupta A.S. (2019). Multiscale Fluctuation-based Dispersion Entropy and its Applications to Neurological Diseases. IEEE Access.

[33] Yao P., Zhou K., Zhu Q. (2017). Quantitative evaluation method of arc sound spectrum based on sample entropy. Mech. Syst. Sig. Process..

[34] Si L., Wang Z., Liu X., Tan C. (2019). A sensing identification method for shearer cutting state based on modified multi-scale fuzzy entropy and support vector machine. Eng. Appl. Artif. Intell..

[35] Zheng J., Dong Z., Pan H., Ni Q., Liu T., Zhang J. (2019). Composite multi-scale weighted permutation entropy and extreme learning machine based intelligent fault diagnosis for rolling bearing. Measurement.

[36] Zhu K., Song X., Xue D. (2014). A roller bearing fault diagnosis method based on hierarchical entropy and support vector machine with particle swarm optimization algorithm. Measurement.

[37] Naik J., Dash P.K., Dhar S. (2019). A multi-objective wind speed and wind power prediction interval forecasting using variational modes decomposition based Multi-kernel robust ridge regression. Renew. Energy.

[38] Lian J., Liu Z., Wang H., Dong X. (2018). Adaptive variational mode decomposition method for signal processing based on mode characteristic. Mech. Syst. Sig. Process..

[39] Liu J., Li Y.-F., Zio E. (2017). A SVM framework for fault detection of the braking system in a high speed train. Mech. Syst. Sig. Process..

